# DNA repair pathways and cisplatin resistance: an intimate relationship

**DOI:** 10.6061/clinics/2018/e478s

**Published:** 2018-08-28

**Authors:** Clarissa Ribeiro Reily Rocha, Matheus Molina Silva, Annabel Quinet, Januario Bispo Cabral-Neto, Carlos Frederico Martins Menck

**Affiliations:** I Departamento de Microbiologia, Instituto de Ciencias Biomedicas, Universidade de Sao Paulo, Sao Paulo, SP, BR; II Instituto de Biofisica Carlos Chagas Filho, Universidade Federal do Rio de Janeiro (UFRJ), Rio de Janeiro, RJ, BR

**Keywords:** Cisplatin, Resistance, DNA Repair, DNA Damage Tolerance

## Abstract

The main goal of chemotherapeutic drugs is to induce massive cell death in tumors. Cisplatin is an antitumor drug widely used to treat several types of cancer. Despite its remarkable efficiency, most tumors show intrinsic or acquired drug resistance. The primary biological target of cisplatin is genomic DNA, and it causes a plethora of DNA lesions that block transcription and replication. These cisplatin-induced DNA lesions strongly induce cell death if they are not properly repaired or processed. To counteract cisplatin-induced DNA damage, cells use an intricate network of mechanisms, including DNA damage repair and translesion synthesis. In this review, we describe how cisplatin-induced DNA lesions are repaired or tolerated by cells and focus on the pivotal role of DNA repair and tolerance mechanisms in tumor resistance to cisplatin. In fact, several recent clinical findings have correlated the tumor cell status of DNA repair/translesion synthesis with patient response to cisplatin treatment. Furthermore, these mechanisms provide interesting targets for pharmacological modulation that can increase the efficiency of cisplatin chemotherapy.

## INTRODUCTION

Nearly five decades after FDA (Food and Drug Administration) approval for the treatment of testicular cancer, cisplatin remains one of the most effective antitumor agents. In fact, in combination with bleomycin and etoposide, cisplatin yields cure rates above 95% 1. In addition to its outstanding success in testicular cancer, a wide variety of solid tumors, such as ovarian, lung, bladder, cervical and head and neck neoplasms, have also been shown to be highly responsive to cisplatin 2. Nevertheless, there is consensus that tumor resistance is the Achilles' heel of cisplatin treatment.

**Figure 1:**
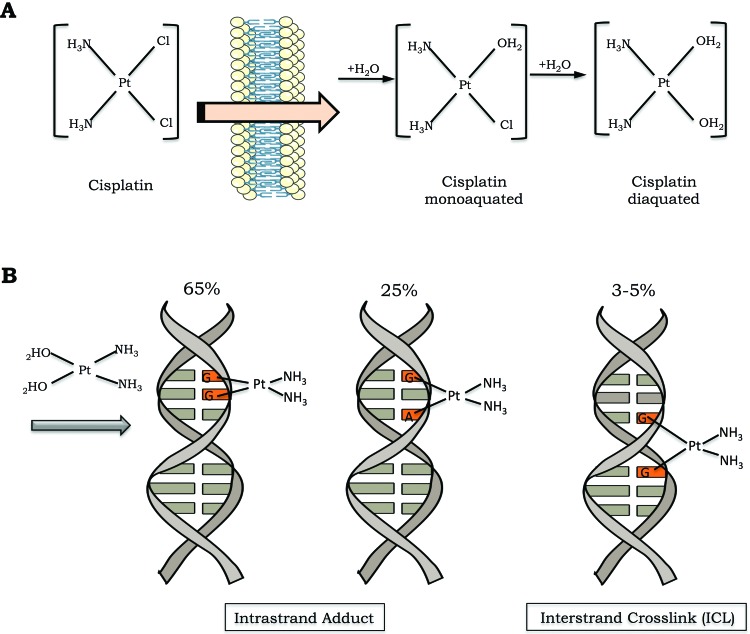
Cisplatin activation and DNA damage induction. A) The cisplatin activation process occurs by exchange of one or two of its chlorides for water molecules (monoaquated and diaquated, respectively). B) Cisplatin can form covalent bonds with DNA. The major DNA lesions are intrastrand DNA adducts and interstrand crosslinks (ICLs). The percentages represent the frequency of each type of DNA damage induced by cisplatin.

Cisplatin is a small and remarkably simple molecule composed of one platinum atom linked to two amides and two chlorides; despite its size, it is a very powerful drug. In conditions of low chloride concentration, as found in the cytosol, cisplatin undergoes a process known as aquation, in which one or two chlorides are replaced with water molecules (Figure 1A). Through the aquation process, cisplatin becomes highly reactive and readily binds to a variety of biomolecules inside the cell 1. In its reactive form, cisplatin covalently binds to DNA bases, forming DNA adducts. Cisplatin particularly reacts with the nucleophilic N7-sites of purine bases, and a double reaction may covalently link purines. When purines are located on the same strand, intrastrand adducts are produced, or alternatively, an interstrand crosslink (ICL) is generated if the purine are on opposite strands 3 (Figure 1B).

Cisplatin-induced DNA adducts block transcription and DNA synthesis, which in turn triggers an intricate intracellular signal transduction cascade in an orchestrated attempt by the cells to eliminate the lesions. The cell cycle is arrested, providing adequate time for DNA repair mechanisms to remove the lesions. In cases of impaired repair or excessive damage, the cells undergo apoptosis 3.

**Figure 2:**
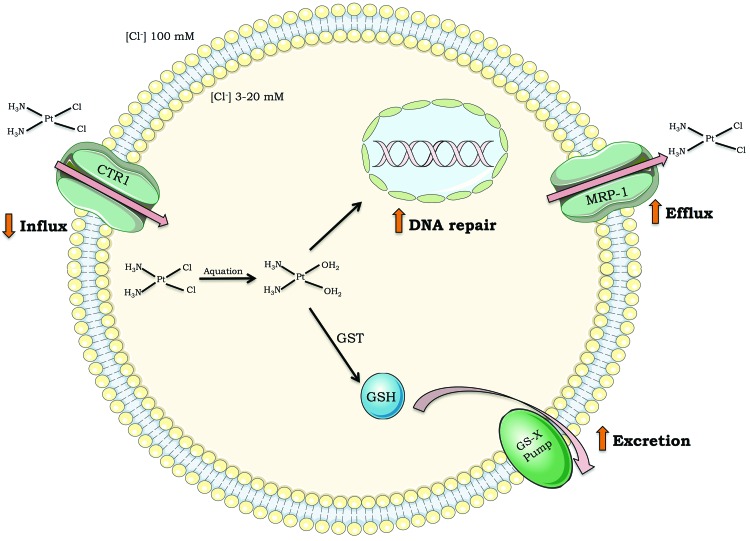
Mechanisms of tumor cell resistance to cisplatin. Drug influx inside cells (i.e., decreased expression of transporter channels); drug efflux outside cells (i.e., increased expression of multidrug transporter channels); drug detoxification (i.e., high levels of glutathione or glutathione S-transferase); DNA repair capacity. Abbreviations: CTR1 (chloride transport receptor 1); GSH (glutathione); GST (glutathione S-transferase); MRP-1 (multidrug resistance-associated protein 1); GS-X pump (ATP-dependent glutathione S-conjugate export pump).

Although cisplatin is highly efficient, intrinsic resistance and resistance acquired during treatment cycles are relatively common and remain a major challenge for cisplatin-based anticancer therapy. There are many ways in which cells block cisplatin from reaching and damaging DNA, including decreasing drug uptake, increasing drug efflux, and inducing drug detoxification by covalent binding to glutathione or metalloproteins 4-7 (Figure 2). However, once cisplatin reacts with DNA, cells must remove or tolerate the lesions in order to resist the treatment effects; otherwise, cisplatin-induced DNA damage will induce massive cell death, including by apoptosis.

Once cisplatin causes many different DNA lesions, most of the major DNA repair systems are involved in removing cisplatin-induced DNA damage. In fact, nucleotide excision repair (NER), mismatch repair (MMR), homologous recombination (HR) and nonhomologous end joining (NHEJ) are involved in repairing cisplatin-induced DNA damage 8. Alternatively, cisplatin-induced DNA damage can be tolerated by simply replicating the damaged molecule through a system known as translesion synthesis (TLS) 9.

In this review, we describe how cisplatin-induced DNA lesions are repaired or tolerated by human cells and discuss the pivotal role of these mechanisms in tumor resistance to cisplatin. Furthermore, we present the most recent clinical findings correlating the DNA repair/TLS status of tumors with the patient response to cisplatin treatment.

## DNA REPAIR PATHWAYS

### Nucleotide excision repair

**Figure 3:**
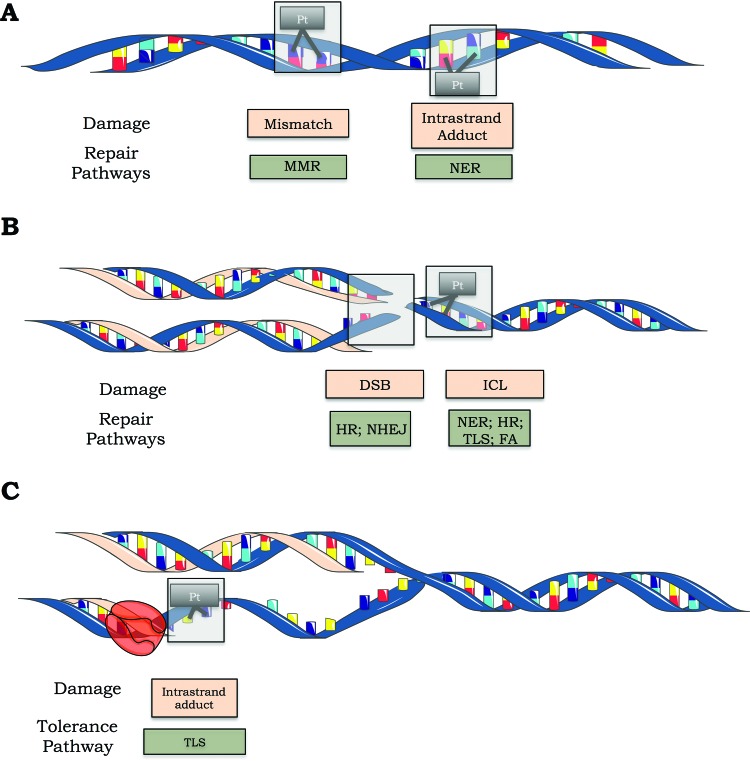
Cisplatin-induced DNA lesions and repair mechanisms. A) The nucleotide excision repair (NER) pathway is responsible for removing cisplatin-induced DNA adducts (such as 1,2 and 1,3 intrastrand adducts), while the mismatch repair (MMR) pathway can recognize but not repair these adducts. B) Double-strand breaks are repaired by homologous recombination (HR) and nonhomologous end joining (NHEJ). The NER, HR, translesion synthesis (TLS) and Fanconi anemia (FA) pathways are involved in ICL repair. C) Schematic representation of lesion bypass of cisplatin-induced intrastrand crosslinks via the TLS pathway.

The bulky DNA adducts generated by cisplatin are mainly repaired by the NER pathway (Figure 3A). This repair mechanism is composed of two subpathways: global genome repair (GGR), which recognizes and repairs damage throughout the genome, and transcription-coupled repair (TCR), which addresses lesions at actively transcribing genes. Proteins in the NER pathway first recognize the lesion: this step is accomplished by the heterodimer XPC-HR23B in GGR and by the blockade of RNA polymerase II elongation, in association with the CSA and CSB proteins, in TCR. The subsequent steps are shared by TCR and GGR and involve double helix strand separation by specific helicases (XPB and XPD, subunits of the TFIIH complex), followed by cleavage of the damaged strand on both sides of the lesion by the endonucleases XPF/ERCC1 and XPG, which cleave the phosphodiester chain at the 5' and 3' ends, respectively, a few nucleotides away from the lesion. Finally, the oligonucleotide (approximately 30 nucleotides long) containing the lesion is excised, and the resulting gap is filled in by DNA polymerase ε using the intact strand as a template 10.

Undoubtedly, cisplatin chemotherapy for testicular cancer, including advanced metastatic testicular cancer, represents a remarkable clinical success. The hypersensitivity of testicular cancer cells to cisplatin reflects the clinical responses, so many studies have been carried out in order to reveal the molecular mechanisms that could possibly explain this phenomenon 11. Data suggest that DNA repair deficiency in testis cells is a determining factor for the high sensitivity of this type of tumor to cisplatin. In fact, testis cell lines had a lower capacity for repairing platinum-DNA adducts than cell lines of bladder lineages, as observed either in the whole genome or in actively transcribed genes 12. Subsequently, the lower DNA repair capacity of testicular cancer cell lines was confirmed by measuring the ability of cell extracts to remove platinum-DNA adducts, and the addition of XPA protein to the cell extracts was sufficient to restore *in vitro* DNA repair capacity 12. Interestingly, XPA was not found to be a rate-limiting protein for NER in UV-irradiated cells 13, which was later also shown to be true for testicular cancer cell lines treated with cisplatin, indicating that low levels of XPA protein are not responsible for increased sensitivity to cisplatin 13. More recently, testicular cancer cell lines were shown to have normal repair of intrastrand adducts and platinum-DNA adducts but reduced levels of ICL repair compared to bladder cancer cell lines. In those studies, low levels of the ERCC1-XPF complex in testis cells correlate well with low ICL repair, and this protein complex seems to be the limiting factor for the repair of these lesions and for cellular cisplatin resistance 14.

The fact that cisplatin has similar efficacy as first-line therapy for other tumors strongly indicates that DNA repair mechanisms play pivotal roles in drug resistance. In ovarian cancer cell lines, early evidence indicated that cisplatin resistance was mediated by a greater capacity to remove cisplatin-induced DNA damage, including ICLs, and greater tolerance of DNA damage 15,16. Later, it was shown that ovarian tumors from treatment naïve patients had similar levels of cisplatin-induced DNA damage, but tumor cells from previously treated patients who showed resistance had an enhanced capacity to remove cisplatin-induced ICLs 17. Moreover, in a set of sensitive and acquired cisplatin-resistant ovarian cell lines, resistance to cisplatin correlated with resistance to UV light, which indicates a role for NER activity in the drug resistance process 18.

Interestingly, the ERCC1/XPF complex seems to be associated with cisplatin resistance in other tumors as well. In fact, early data indicated that low expression of ERCC1 or XPF was associated with increased sensitivity to cisplatin in both cell lines and tumor tissues. In ovarian cancer cells, ERCC1 expression was found to be induced after cisplatin treatment 19, and this induction correlated with increased capacity for the repair of cisplatin-induced DNA damage 20. On the other hand, low ERCC1 mRNA levels in primary gastric adenocarcinoma and lung cancer showed a positive association with improved survival of patients treated with cisplatin in combination with fluorouracil or gemcitabine 21,22.

In addition to DNA adducts, which are the main substrates for the NER pathway, ICL lesions are also generated by cisplatin. There is abundant evidence that NER proteins also play an important role in the ICL repair pathway. Among NER proteins, XPD, XPF, XPG and ERCC1 participate in ICL repair. Thus, low levels of one of these genes could, in principle, lead to even lower DNA repair capacity, which in turn could induce more cell death. In agreement with this hypothesis, XPD protein was shown to interact with the HR protein Rad51 and increase the rate of ICL removal 23.

Moreover, ERCC1 participates in both the NER and HR pathways, increasing the importance of this protein for DNA damage processing. In fact, ERCC1 deficiency leads to higher sensitivity to cisplatin than does XPA deficiency 24. In a study of 761 lung cancer patients, Olaussen et al. 25 observed that almost half of them were negative for ERCC1 expression and that cisplatin treatment significantly prolonged the survival of patients with ERCC1-negative tumors but not that of those with ERCC1-positive tumors. Similarly, gastric adenocarcinoma patients with low ERCC1 levels showed a greater benefit from cisplatin treatment than those with high ERCC1 levels 21.

From the initial findings, consistent data indicate a good correlation between the ERCC1/XPF nuclease, and NER in general, and tumor resistance to cisplatin, although some discrepant results of a lack of correlation have also been reported 26. Thus, the use of ERCC1 (and/or other NER genes) as predictors of tumor prognosis may be an important clinical strategy. Moreover, recent data indicated that ERCC1 silencing by RNA interference may provide a means to potentiate cisplatin-induced tumor cell killing 27. These data are not definitive and do not discount the relevance of other DNA repair/tolerance pathways in cisplatin resistance.

### Mismatch repair

The MMR pathway is the DNA repair mechanism responsible for correcting single-strand DNA (ssDNA) errors, such as mismatches or insertions/deletions generated during replication. Similar to NER, MMR consists of recognition, excision, resynthesis and ligation of the newly synthetized strand. The first step of the MMR pathway is accomplished by two heterodimers, namely, the MutSα (formed by MSH2-MSH6) and MutSβ (MSH2-MSH3) heterodimers, which form a complex with MutL. In a process regulated by ATP, this complex slides up and down double-strand DNA until it encounters proliferating cell nuclear antigen (PCNA) and replication factor C (RFC), replication machinery proteins. Then, the exonuclease EXO1 cleaves the DNA daughter strand harboring nucleotide mismatches, and high-fidelity replicative polymerases (Polδ and Polε) fill the gap after oligonucleotide excision. Finally, DNA ligase rejoins the DNA sequence 28.

In addition to correcting postreplicative errors, MMR also recognizes lesions caused by alkylating agents, including cisplatin, that induce postreplicative mispairing, mainly involving G/T (Figure 3A). Nonetheless, the MMR pathway is unable to repair these lesions because MMR replaces the base opposite the cisplatin adduct, thus keeping the source of the mismatch intact. Since it ultimately fails to fully repair the lesion, the MMR pathway will start this process again, leading to a so-called futile cycle. This constant procedure of cutting and mending the DNA strand can eventually lead to the formation of double-strand breaks (DSBs) and the activation of DNA damage signaling factors, including p53, ataxia telangiectasia mutated (ATM) and ataxia telangiectasia related (ATR), ultimately triggering apoptosis. In addition to the futile cycle, MMR proteins may shield cisplatin adducts by inhibiting NER proteins from repairing them, therefore allowing adducts to persist and consequently enhancing cisplatin lethality 28. Thus, contrary to deficiencies in NER, those in MMR mechanisms are thought to be involved in tumor resistance to cisplatin, which has been corroborated by early studies demonstrating that MMR deficiency confers resistance to cisplatin in *Escherichia coli* 29.

Deficiency in the MMR pathway can occur either by epigenetic modification (gene silencing due to promoter methylation) or gene mutation (leading to protein dysfunction). The most common mechanism for MMR deficiency is CpG island hypermethylation of the MLH1 promoter on both alleles, and an alternative mechanism is genetic inheritance. In the latter case, the MSH2, MLH1, PMS2 or MSH6 genes are mutated, as seen in Lynch syndrome 30-32. In MMR-deficient cells, mismatched nucleotides accumulate, generating microsatellite instability. Thus, impairment of this DNA repair pathway is highly associated with several types of cancer, including hereditary nonpolyposis colon cancer and colorectal carcinoma. In addition, it is estimated that MMR deficiencies are present in approximately 17% of all primary tumors 33,34.

Promoter hypermethylation of MMR genes was shown to actually increase after cisplatin treatment and, as a consequence, contribute to the acquisition of resistance 35. Accordingly, the cisplatin resistance of human ovarian or colon cancer cell xenografts, which have hypermethylation of the MLH1 gene promoter, grown in nude mice was overcome when the cells were inoculated with the demethylating agent 2'-deoxy-5-azacytidine (DAC) and the mice were treated with cisplatin 36.

MMR deficiency has been implicated in many studies as a relevant pathway for cisplatin resistance. For instance, the loss of MLH1 in colon cancer cells resulted in a 2-fold increase in cisplatin resistance 37. Similar results were reported in the endometrial cancer cell line HEC59, which is deficient in MSH2, in which a 1.8-fold increase in cisplatin resistance was observed 38. In addition, genetic complementation of MLH1 in deficient cells restored cisplatin sensitivity 39.

However, it is important to note that the changes in cisplatin sensitivity in cell lines in culture are relatively modest, and there are many conflicting reports regarding the clinical impacts of MMR functional status on tumor cells and patient survival. In an analysis of NSCLC (non-small cell lung carcinoma) patients, Hsu et al. 40 showed that hypermethylation of the promoter regions of the hMSH2 and hMLH1 genes was associated with poor patient survival. Similar results have been reported in ovarian and breast cancer patients 41,42. On the other hand, Cooper et al. 43 showed no correlation between MMR proficiency and patient survival rate. Moreover, low levels of MSH2 were correlated with the long-term survival of lung cancer patients treated with cisplatin 44. Further studies are therefore necessary to elucidate the role of MMR in cisplatin resistance in cancer.

### DSB and interstrand crosslink (ICL) repair

The HR and NHEJ pathways repair DNA DSBs, which can be generated by the well-known agents ionizing radiation and free radicals. In addition, the ICL repair process results in DSBs, which are considered the most hazardous type of DNA damage for a cell. This fact may be explained by the difficulty in repairing this type of damage, since unlike all other lesions that rely on an intact strand to serve as a template for repair, the integrity of both DNA strands is lost in DSBs 45.

In the event of a DSB, the HR mechanism uses a homologous DNA sequence as a template to correct the broken strand. Briefly, after DSB induction, a complex cascade of reactions is triggered in order to arrest the cell cycle and recruit DNA repair factors. The ATM kinase acts a DSB sensor that leads to G1 arrest mediated by p53 phosphorylation. Subsequently, ATR and DNA-PK_cs_ (DNA-dependent protein kinase) are activated. ATR controls the response to a large spectrum of DNA damage, including lesions that interfere with DNA replication, such as ssDNA breaks (SSBs) and stalled DNA replication forks, while ATM and DNA-PK_cs_ play an important role in responses to DSBs 46. Finally, histone H2AX is phosphorylated at sites of DNA lesions, enabling the recruitment of DNA repair proteins 47. Since the HR pathway requires DNA strand homology to repair DSBs, it generally works in only proliferating cells after sister chromatids are synthetized in the S phase of the cell cycle 48.

Contrary to error-free HR, the NHEJ pathway is considered an error-prone DNA repair mechanism because it may delete some nucleotides during the ligation of DSBs 49. In this pathway, DSBs are identified by Ku70/Ku80 heterodimers. Then, DNA-PK_cs_ binds to DNA, and the Artemis-DNA-PK_cs_ complex functions as an endonuclease, cleaving several nucleotides at the DSB site. To complete the DNA repair, resynthesis is accomplished by DNA polymerases μ and λ, and DNA ligase IV restores the phosphodiester backbone. Importantly, DSB repair in G0/G1 phase is accomplished predominantly by NHEJ. In contrast, in order to repair DSBs, HR requires a sister chromatid to serve as a template to synthesize the portion of the strand undergoing repair. Therefore, HR plays a major role in repairing lesions in cells in S/G2 phase. This distinction has an obvious impact on the selection of the repair strategy in proliferating *versus* postmitotic cells 50.

ICLs are extremely cytotoxic, especially for proliferating cells, because ICLs covalently bind the two strands of the DNA helix together, which prevents strand separation and thereby inhibits vital cellular processes such as DNA replication and transcription 51. Invariably, replication of DNA containing ICLs induces DSBs, possibly due to collapse of the replication fork. The repair of ICLs is incredibly complex and involves numerous pathways, including NER, TLS, HR and Fanconi anemia (FA) proteins 52 (Figure 3B). Briefly, the XPF/ERCC1 nuclease complex cleaves one of the crosslinked strands, generating an intrastrand dinucleotide adduct 53. Subsequently, recombination proteins, including Rad51, Rad52, XRCC2, XRCC3, and RPA, generate a structure called a Holliday junction that is resolved by the MUS81/MMS4 complex 54. In addition to this pathway, the TLS process performed by the error-prone DNA polymerase η plays a significant role in ICL repair 52,55. Furthermore, corroborating the fact that cells from FA patients are sensitive to ICL-inducing agents, there is evidence indicating that the FA/BRCA pathway participates in both ICL and DSB repair by HR 56.

ICL repair in human cells is greatly dependent on the cell cycle status, which means that the genetic requirements for repair and the repair outcomes for these particular types of lesions are quite different in dividing and nondividing cells. In this section, however, assuming that malignant tumors ultimately increase in size through uncontrolled proliferation of abnormal cells, we have decided to focus exclusively on insights from current investigations concerning the mechanism of ICL repair in dividing cells.

ICLs represent only <5% of total DNA platination. Nevertheless, this type of DNA lesion is one of most deadly induced by cisplatin because the information encoded by the complementary strand cannot be accessed while DNA strands remain crosslinked. The introduction of these lesions in DNA can lead, therefore, to impaired replication and transcription and can hinder any other process requiring strand separation, thereby triggering cell death. There is a large amount of experimental evidence suggesting that in mammalian cells, genomic ICLs are targeted by the so-called FA/BRCA pathway, which might explain why FA patients are particularly affected by alkylating agents at the cellular level.

Normal replication or cell exposure to several ICL damaging agents triggers a sequence of molecular events leading to activation of the FA/BRCA pathway. The early event in FA pathway activation is the formation of the FA core complex, which monoubiquitinates the FANCD2 and FANCI proteins, a crucial step in FA/BRCA pathway activation. The innate recognition and early processing stages of ICL in dividing cells seem to be mediated by two partially redundant signaling pathways involving FA proteins, RPA, PCNA and BRCA2, which encounter the lesion at the replication fork and collapse it during S phase, as well as components of the NER pathway, such as XPA and XPC-HR23B, at the G1 phase of the cell cycle. Therefore, ICL-induced replication arrest during S phase activates the assembly of the FA core complex, as well as the monoubiquitination of FANCD2/FANCI, which is translocated to the chromatin, where it binds other repair factors, such as BRCA1, γ-H2AX, Rad51 and PCNA. Once a set of DNA damage recognition proteins is established, the endonuclease complex ERCC1-XPF is immediately recruited to the site of DNA damage, preparing the next step of the repair process.

Downstream events of ICL repair require the coordinated and hierarchical action of three classical pathways, namely, the NER, TLS and HR pathways. Replication fork stalling is also recognized by the FANCM–FAAP24–MHF complex, which recruits the FA core and Bloom helicase complex to chromatin and activates ATR/ATRIP signaling through the binding of RPA to ssDNA. The first incision at the damaged strand is accomplished by active Mus81-Eme1 endonuclease, thus converting the stalled replication fork to a DSB. As already mentioned, ICLs covalently link two bases on complementary strands of DNA; thus, after the first 3' incision, the lesion remains attached to both strands. However, after the second 5' incision, carried out by XPF-ERCC1, a stable flap is formed, and the lesion is properly removed from the lagging strand template, allowing a translesion polymerase to extend the leading strand synthesis past the removed lesion and thereby generating a double-stranded structure appropriate for sustained targeted HR. In addition to HR, the NER system may also be involved in the repair of the broken sister chromatid and the removal of the adduct remaining attached to the parental strand.

Since abrogation of at least five interlinked pathways (FA, NER, Bloom, ATR/ATRIP signaling and HR) renders cells particularly vulnerable to ICL-inducing anticancer drugs, it seems logical to suppose that the genes integrating these pathways are potentially implicated in the outcome of cancer treatment. Burkitt and Ljungman 57 reported the validation of a clinical protocol for the prognosis and treatment optimization of head and neck cancer cells by comparing the functional status of the FA/BRCA pathway and cisplatin sensitivity. As a result, recruitment of FANCD2 to nuclear foci was significantly correlated with greater sensitivity to cisplatin. Furthering this line of research, the same authors 58 reported that the histone deacetylase inhibitor phenylbutyrate sensitizes human cells to cisplatin. Inhibition of the FA/BRCA pathway through BRCA1 downregulation seems to be responsible for the sensitization, since pretreatment with phenylbutyrate increased cisplatin sensitivity in cisplatin-resistant head and neck cancer cells while simultaneously decreasing cisplatin-induced FANCD2 foci formation.

There has been a considerable increase in the body of evidence showing a link between virtually all HR-related proteins and the mechanism of either inherent or acquired cellular resistance to cisplatin. The identification of the MCM8-MCM9 complex, which promotes RAD51 recruitment to damage sites in mammalian cells, thereby allowing the high-throughput assessment of DNA repair through HR, strongly supports this idea. Depletion of MCM8 or MCM9 in human cancer cells or MCM9 loss of function in mouse embryo fibroblasts sensitizes cells to ICL-inducing agents, such as cisplatin 59.

## CISPLATIN-INDUCED DNA DAMAGE TOLERANCE

### Translesion synthesis

Cisplatin-induced intrastrand adducts are bulky lesions that interfere with DNA replication machinery by arresting replicative DNA polymerases. The prolonged stalling of replication forks can result in the formation of DNA DSBs, and such deleterious damage can lead to gross DNA rearrangements or cell death 60. To avoid the collapse of arrested replication forks, DNA damage can be tolerated by TLS 61 (Figure 3C). TLS is performed by specialized polymerases (TLS Pol) of the Y (Polymerase η (Polη), Polι, Polκ and Rev1) and B families (such as Polζ) that have a broad catalytic site and lack proofreading activity, enabling it to bypass the lesion in an accurate or mutagenic manner 62.

Polη is responsible for the TLS of a number of bulky DNA adducts, including UV-induced cyclobutane pyrimidine dimers. *In vitro* studies have shown that human Polη is also able to replicate cisplatin-GG adducts 63 by inserting a correct dCMP opposite the 3'dG of cisplatin-GG and continuing chain elongation; Polη can also incorporate an incorrect nucleotide, and in that case, it is not able to extend the primer 64. Moreover, structural and biochemical analyses have shown that Polη inserts the correct nucleotide opposite the first G of cisplatin-GG, but it is less efficient and promiscuous in the bypass of the 5'dG 65,66. Human cells lacking Polη are more sensitive to cisplatin treatment than wild-type cells or the same cell lines complemented with functional Polη, demonstrating the physiological relevance of the ability to bypass cisplatin-induced intrastrand adducts by Polη 67. Importantly, although the sensitivity of XP-V cells to cisplatin was comparable to that of NER-deficient XP-A cells, Polη was shown to be essential for overcoming cisplatin-induced S phase arrest and to colocalize with monoubiquitinated PCNA 67; this posttranslational modification of the clamp occurs in the early steps of TLS. Additionally, upon cisplatin treatment, Polη was strongly induced in a P53-dependent manner 68, and it was necessary for the progression of replication forks in human cells 69. These findings demonstrate the role of Polη in the TLS of cisplatin-induced intrastrand adducts in the human genome.

Moreover, another TLS polymerase, Polζ, which is formed by at least two subunits, Rev3L and Rev7, is also involved in the bypass of cisplatin-induced intrastrand adducts. Indeed, transformed mouse cells lacking Polζ were shown to be more sensitive to cisplatin than their Polζ-proficient counterparts 70. Additionally, knockdown of the catalytic subunit of Polζ, Rev3L, rendered human cells more sensitive to the cytotoxic effects of cisplatin 71. In addition to Polη and Polζ, TLS Pol Rev1 was also shown to be important for the bypass of cisplatin-induced intrastrand adducts 72. Despite its ability to insert one dCMP opposite the DNA damage, Rev1 plays a noncatalytic role in TLS by scaffolding TLS polymerases, such as Polζ, to DNA 73.

A more recent study showed that the TLS of cisplatin-induced bulky adducts is in fact dictated by a two-Pol mechanism 74. By using plasmids carrying specific single-site lesions and knocking down TLS polymerases, the authors demonstrated that Polη or Polκ inserts the first nucleotides in front of the cisplatin-GG, followed by extension of the DNA primer by Polζ. It is worth noting that Polη activity results in error-free translesion replication, while Polκ in combination with Polζ results in error-prone TLS past cisplatin adducts 74. In agreement, Wei Yang's group was the first to purify human Polζ and demonstrated *in vitro* that the bypass of cisplatin-GG requires Polη to insert dCMP opposite the 3'dG and Polζ to extend the primers 75.

Because the tolerance of intrastrand crosslinks counteracts cisplatin-induced cell death, TLS Pol expression has been correlated with resistance to this drug. The analysis of sixty-four mucosal-derived head and neck squamous cell carcinomas (HNSCCs) revealed that Polη expression was elevated in 67% of the samples and that low Polη levels were significantly associated with a high complete response rate after cisplatin treatment 76. A recent report showed elevated Polη expression in ovarian cancer stem cells isolated from ovarian cancer cell lines and primary tumors 77. In this study, knockdown of Polη blocked cancer stem cell enrichment by enhancing cisplatin-induced apoptosis.

The data indicate that TLS mediated by Polη is involved in the survival of cancer stem cells upon cisplatin treatment, and the authors propose targeting Polη as a strategy to increase the efficiency of this chemotherapeutic. Moreover, Polζ expression has also been reported as a predictor of cisplatin resistance. The analysis of Rev3L expression revealed higher protein levels in human glioma than in normal brain tissues 78. The engineered overexpression of Rev3L in a glioma cell line attenuated cisplatin-induced apoptosis, while depletion of this protein increased cell sensitivity to this drug. Additionally, the analysis of 123 patients with squamous cell cervical carcinoma who had adjuvant chemoradiation therapy after radical surgery revealed that Polζ expression is a significant predictor of recurrence and that positive Polζ expression is associated with the depth of cervical stromal invasion 79. In agreement, it was recently reported that Polζ expression is higher in cervical cancer than in normal tissue; in cervical cancer, the depletion of Rev3L increased cisplatin sensitivity, while Rev3L overexpression conferred cisplatin resistance by decreasing cisplatin-induced apoptosis 80. These authors also propose targeting these TLS Pols to counteract cisplatin resistance.

On the other hand, because TLS Pols can be error-prone when they bypass intrastrand adducts, they can contribute to the acquisition of cisplatin resistance. In human fibroblasts, cisplatin induced Rev3L expression in a concentration- and time-dependent manner, and Rev3L depletion decreased HR and TLS, not intrastrand adduct repair, upon cisplatin treatment 71. These data strongly indicate that in addition to its role in cell survival upon cisplatin treatment, Polζ is involved in genetic instability and the subsequent generation of drug-resistant variants in the surviving population 71. In human colon carcinoma cell lines, Rev3L depletion decreased mutagenicity and acquired resistance to cisplatin. The enhanced cisplatin-induced mutagenicity observed in cancer cells defective for MMR was completely dependent on Polζ 81. Therefore, Polζ plays a central role in mutagenicity and cisplatin resistance in cancer cells, particularly in MMR-deficient cancer cells.

Rev1 was also shown to be involved in the acquisition of cisplatin resistance. Overexpression of human Rev1 in human ovarian carcinoma enhanced cisplatin-induced mutagenicity and cisplatin resistance after sequential cycles of drug exposure that mimic clinical schedules of drug administration 82. In agreement, suppression of Rev1 in a mouse model of B cell lymphoma inhibited cisplatin-induced mutagenesis and acquired resistance upon repeated cycles of tumor engraftment and treatment 83.

Therefore, inhibition of TLS polymerases can have a dual anticancer effect, sensitizing the tumors to the drug and limiting the emergence of tumor chemoresistance. Indeed, Xie et al. 83 evaluated the effect of Rev3L depletion on the response to cisplatin *in vivo* in aggressive late-stage lung carcinoma; the tumors exhibited pronounced sensitivity to the treatment, followed by a significant increase in the overall survival of treated mice. Additionally, Rev3L-depleted cells presented diminished mutagenesis, a process that is highly correlated with the induction of secondary malignancies following chemotherapy.

## DNA REPAIR MODULATION STRATEGIES

DNA repair and DNA damage tolerance play crucial roles in the response to cisplatin treatment, making DNA damage response and repair proteins tempting therapeutic targets to modulate chemoresistance, sensitize tumor cells to cisplatin and enhance chemotherapeutic efficiency. However, many of the agents developed so far have shown low target specificity and have failed to reach the clinic.

Due to the success of PARP inhibitors in the treatment of BRCA-deficient tumors, there has recently been renewed interest in developing small molecule inhibitors of DNA repair proteins (Table 1) 84. In NER, the main targets are the RPA and XPA proteins and the ERCC1-XPF heterodimer. An *in silico* screen identified a compound—F06/NERI02 (NSC130813)—that, despite its modest *in vitro* affinity for XPF (30 µM), blocked the ERCC1-XPF interaction and increased cisplatin sensitivity 85. Other *in silico* studies identified several catechol and hydroxyl-imide/pyridine/pyrimidinone agents as ERCC1-XPF inhibitors, such as E-X PPI2 and E-X AS7. E-X PPI2 significantly reduced ERCC1-XPF heterodimer levels in ovarian cancer cells, blocked NER activity (IC_50_ = 20 µM) and enhanced melanoma cell sensitivity to cisplatin. E-X AS7 displayed greater specificity for inhibiting NER activity (IC_50_ = 2 µM) and increased the cisplatin sensitivity of NER-proficient, not NER-deficient, human cells 86,87. Another compound, NERI01, inhibited the interaction between ERCC1 and XPA, sensitizing colon cancer cells to UV irradiation, but had a weaker effect on cisplatin sensitivity 88.

Another promising target is replication protein A (RPA), which plays important roles in both the NER and HR pathways and is overexpressed in a number of tumors. RPA is a ssDNA binding protein that supports NER-catalyzed repair of bulky adduct DNA damage. The compound TDRL-551 showed a synergistic effect with cisplatin due to its ability to block the RPA-DNA interaction 89. Another mechanism of inhibition involving covalent modification of RPA was discovered with the isoborneol haloacetate MCI13E, which greatly potentiated the cisplatin-induced death of lung cancer cells 90.

In contrast to deficiencies of other repair pathways, deficiency of the MMR pathway improves damage tolerance, contributing to increased mutagenicity, tumor heterogeneity and chemoresistance. Therefore, there is no interest in developing inhibitors of MMR proteins. Since MMR activity increases cell sensitivity to cisplatin, the research focus has been on compounds that restore MMR function. In many cancers, the *hMLH1* gene promoter is hypermethylated, causing a reduction in MLH1 protein levels. Cell-based studies have shown that a fluoropyrimidine derivative, 5-fluoro-2-deoxycytidine (FdCyd), and a cytidine analog, 2'-deoxy-5-azacytidine (decitabine), have potential utility in reversing this hypermethylation, restoring MMR functionality and consequently sensitizing cells to cisplatin 91,92.

Due to the involvement of several different pathways in the repair of ICLs, there are many proteins that can be inhibited to induce sensitization to cisplatin. One of these targets is the kinase ATR, which is involved in the DNA damage response (DDR) signaling pathway and in the HR pathway, for which there are two inhibitors in phase 1 and 2 trials (VX-970 and AZD6738, respectively). VX-970 inhibits ATR (IC_50_ = 19 nM) and induces a 10-fold increase in the sensitivity to cisplatin in half of the cell lines tested; it also significantly improved the responses to cisplatin in xenograft models derived from primary human NSCLC samples 93. Accordingly, AZD6738, an orally active and bioavailable inhibitor, showed strong synergy with cisplatin in lung cancer cell lines 94.

DSBs are formed during the process of repairing ICLs, and these highly toxic lesions can be repaired by either the HR or NHEJ pathway. For the NHEJ pathway, there are two highly selective DNA-PK_cs_ inhibitors, NU7026 and NU7441, that showed synergy with cisplatin in BRCA1-deficient breast cancer cell lines 95. For the HR pathway, one of the strategies involves inhibiting DNA strand exchange activity by disrupting the ability of Rad51 to bind ssDNA. The compound B02 has such activity; it inhibits HR and increases cancer sensitivity to several DNA damaging agents, including cisplatin 96.

The TLS pathway is another important step in the completion of ICL repair. The compound T2AA has been demonstrated to enhance cisplatin cytotoxicity by inhibiting the interaction between mono-ubiquitinated PCNA and REV1, resulting in blockade of ICL repair 97. Two compounds, named compound 4 and 5, that inhibit REV1, thereby sensitizing human cancer cells to cisplatin and decreasing cisplatin-mediated mutagenesis, were recently developed. These compounds showed no activity in a *Rev1^-/-^* MEF cell line, indicating that they are specific for Rev1 98.

The clinical benefit of cisplatin as an antitumoral agent is unquestionable. However, tumor resistance to cisplatin remains a major challenge, and there is clearly an urgent need to improve the therapeutic protocol. Certainly, cisplatin binding to DNA causes the most damage, as this is the main mechanism by which it induces cell death. Once DNA is damaged, the cells must either repair or tolerate the lesions. Thus, DNA repair and tolerance systems are imperative for tumor cell survival and resistance to cisplatin. Therefore, targeting the different DNA repair processes is a tempting therapeutic strategy to enhance the anticancer effects of cisplatin, hopefully with clinical benefits to patients and improvements in their quality of life.

## AUTHOR CONTRIBUTIONS

Rocha CR, Molina MS, Quinet A and Cabral-Neto JB contributed to study conception and manuscript writing. Menck CF critically reviewed the manuscript.

## Figures and Tables

**Table 1:** Summary of DNA repair inhibitors used in combination with cisplatin.

Pathway	Target	Compound	Ref
		F06/NERI02	(85)
	ERCC1-XPF	E-X PPI2	(87)
NER		E-X AS7	(87)
	ERCC1-XPA	NERI02	(88)
	RPA-DNA	TDRL-551	(89)
	RPA	MCI13E	(90)
MMR	MLH1	FdCyd	(91)
		Decitabine	(92)
DDR	ATR	VX-970	(93)
		AZD6738	(94)
NHEJ	DNA-PKcs	NU7026	(95)
		NU7441	(95)
HR	Rad51-ssDNA	B02	(96)
TLS	PCNA	T2AA	(97)
	Revl	Compound 4/5	(98)
